# Inter-Relationships between Frailty, Sarcopenia, Undernutrition and Dysphagia in Older People Who Are Admitted to Acute Frailty and Medical Wards: Is There an Older Adult Quartet?

**DOI:** 10.3390/geriatrics5030041

**Published:** 2020-06-30

**Authors:** David Smithard, Dharinee Hansjee, Darrien Henry, Laura Mitchell, Arjun Sabaharwal, Jo Salkeld, Eirene Yeung, Osman Younus, Ian Swaine

**Affiliations:** 1 Department Geriatric Medicine, Lewisham and Greenwich NHS Trust, London SE13 6LH, UK; dharinee.hansjee@nhs.net (D.H.); lauramitchell6@nhs.net (L.M.); Arjun.sabharwal@nhs.net (A.S.); Osman.younus@nhs.net (O.Y.); 2 School of Health Science, University of Greenwich, London SE9 2UG, UK; I.L.Swaine@greenwich.ac.uk; 3 Department Medicine, Royal Halamshire Hospital, Sheffield S10 2JF, UK; darrien.henry@nhs.net; 4 Department Medicine, St Thomas’ Hospital, London SE1 7EH, UK; jo.salkeld1@nhs.net; 5 Department Acute Medicine, King’s College Hospital, London SE5 9RS, UK; Eirene.yeung1@nhs.net

**Keywords:** dysphagia, nutrition, frailty, sarcopenia

## Abstract

*Introduction*: With increasing age the prevalence of frailty, sarcopenia, undernutrition and dysphagia increases. These are all independent markers of outcome. This study explores the prevalence of these four and explores relationships between them. *Methods*: A convenience sample of 122 patients admitted to acute medical and frailty wards were recruited. Each was assessed using appropriate screening tools; Clinical Frailty Score (CFS) for frailty, SARC-F for sarcopenia, Nutritional Risk Tool (NRT) for nutritional status and 4QT for dysphagia. *Results*: The mean age of the participants was 80.53 years (65–99 years), and 50.37% (68) were female. Overall, 111 of the 122 (91.0%) reported the presence of at least one of the quartet. The median CFS was 5 (1–9), with 84 patients (68.9%) having a score of ≥5 (moderate or severely frail); The median SARC-F was 5 (0–10), with 64 patients (52.5%) having a score of ≥5; The median NRT was 0 (0–8) and 33 patients (27.0%) scored ≥ 1. A total of 77 patients (63.1%) reported no difficulty with swallowing/dysphagia (4QT ≥ 1) and 29 (23.7%) had only one factor. Sixteen patients (13.1%) had all four. There was a significant correlation between nutritional status and dysphagia, but not with frailty or sarcopenia. There were significant correlations between frailty and both sarcopenia and dysphagia. *Conclusions*: In our sample of acute medical and frailty ward patients, there was a much higher prevalence than expected (91%) of either: frailty, sarcopenia, undernutrition or dysphagia. The prevalence of all four was present in 13% of patients. We suggest that frailty, sarcopenia, nutritional risk and dysphagia comprise an “Older Adult Quartet”. Further study is required to investigate the effect of the “Older Adult Quartet” on morbidity and mortality.

## 1. Introduction

The average age of the world’s population is increasing. The aetiology of this is both due to increased survival into adulthood and a greater lifespan. The proportion of people >65 years is significant in many countries including the UK (up to 15%) and Japan (up to 27%) [1,2]. Of the older population, the greatest expansion is seen in the very old (>85 years). Great age is accompanied by many social and health concerns which result in increased dependency and the accumulation of multiple long-term conditions.

In recent years, frailty, sarcopenia and dysphagia have been identified as geriatric syndromes/giants [3,4] and are thought to be the most significant contributors to hospitalisation in older adults. It is recognised that there is a complex inter-relationship between the quartet of frailty, sarcopenia, dysphagia and nutrition. The inter-relationships between these infers that a change in one will result in a cascade of events influencing the other three. Frailty is associated with weight loss, fatigue and slowing up, and can be classified as mild, moderate or severe [5,6]. When frailty is deemed severe, life expectancy is short [5].

Identifying frailty is made difficult by the lack of a universally accepted definition and agreed measure of frailty. There are at least two popular definitions of frailty; one definition is “the accumulation of deficits” [6] and the other is based on “functional decline” [5]. Part of the problem relates to the way frailty is measured, because different scales do not assess the same aspects of frailty. Therefore, it is possible to be assessed as “moderately frail” on one and “severe” on another [7]. In the UK, the Clinical Frailty Score (CFS) is commonly used within frailty services and in primary care. The electronic frailty index (eFI) is also used [8]. The CFS rates people on a categorical scale, where 1 = no frailty, 8 = severe frailty, and 9 = terminal care.

Sarcopenia is a term that describes the presence of a reduced muscle mass, muscle weakness, loss of function and fatigue [9]. Sarcopenia may be primary, where no underlying disease process can be identified or secondary to a chronic disease process (heart failure, COPD) [9]. The prevalence of sarcopenia will vary with age and has been reported to be 30% in those over the age of 85 years [10]. SARC-F is a simple screening tool for sarcopenia [11] and has five domains, each scoring between 0 and 2 to give a maximum score of 10. The higher the score, the more likely it is that sarcopenia is present. Studies have demonstrated that the SARC-F demonstrates a high specificity for the presence of sarcopenia [12]. There are also objective measures that can be used, including hand grip strength, bio-impedance and the timed up and go test.

Dysphagia is a common problem in frail older people. Dysphagia occurs when there is difficulty moving food or liquid from the mouth to the stomach. Carrion et al. reported its prevalence of 47.4% on an acute geriatric unit in Barcelona. The same group also reported that 55% of people with dementia who were admitted to hospital have dysphagia [13,14], The prevalence is even higher if there is concomitant pathology such as stroke or Parkinson’s Disease [15]. Dysphagia can be screened for by using one of the many swallowing screens. Most swallow screens have been validated in the management of stroke. Lately, Tsang et al. [16], and Uhm et al. [17] have published short questionnaires that do not rely on the use of aids (water, teaspoon and cup); these screens have shown promising results in identifying the presence of dysphagia.

Where there is no underlying pathological disease process, contributing to dysphagia, the term presbyphagia is used. Where dysphagia occurs in the context of sarcopenia, the term sarcopenic dysphagia is used [18]. However, reports in the literature have not shown a direct link between peripheral sarcopenia as assessed by handgrip strength and the presence of dysphagia. It is important to identify dysphagia and sarcopenia in frail older people admitted to hospital, so that appropriate management can be implemented, including referral to a speech and language therapist and a dietitian. Frailty and sarcopenia are associated with a pro-inflammatory state and impaired immunity, exposing people to an increased risk of infection and general decline. As a consequence, those who are most frail will be recurrently admitted to hospital.

Frailty, sarcopenia, nutritional risk and dysphagia are all common in older people and the presence of and management of each one impinges directly or indirectly on the others. As part of a Quality Improvement Series of projects to improve dysphagia management on an Acute Medical/Frailty Ward, we have looked at the relationship between sarcopenia, frailty, nutrition and dysphagia.

## 2. Methodology

A convenience sample of older frail adults admitted to acute frailty and medical wards, were approached to take part, by members of the clinical team (O.Y., D.H., L.M., E.Y. and J.S.) during the post-acute phase of their admission. All were screened for the presence of dysphagia, sarcopenia, frailty and poor nutrition, using screening tools that are used in the day-to-day clinical assessments of patients. Verbal consent was obtained from all participants. This work was part of a Quality Improvement project. Approval was granted by departmental and Divisional Governance Committees.

**Figure 1:**
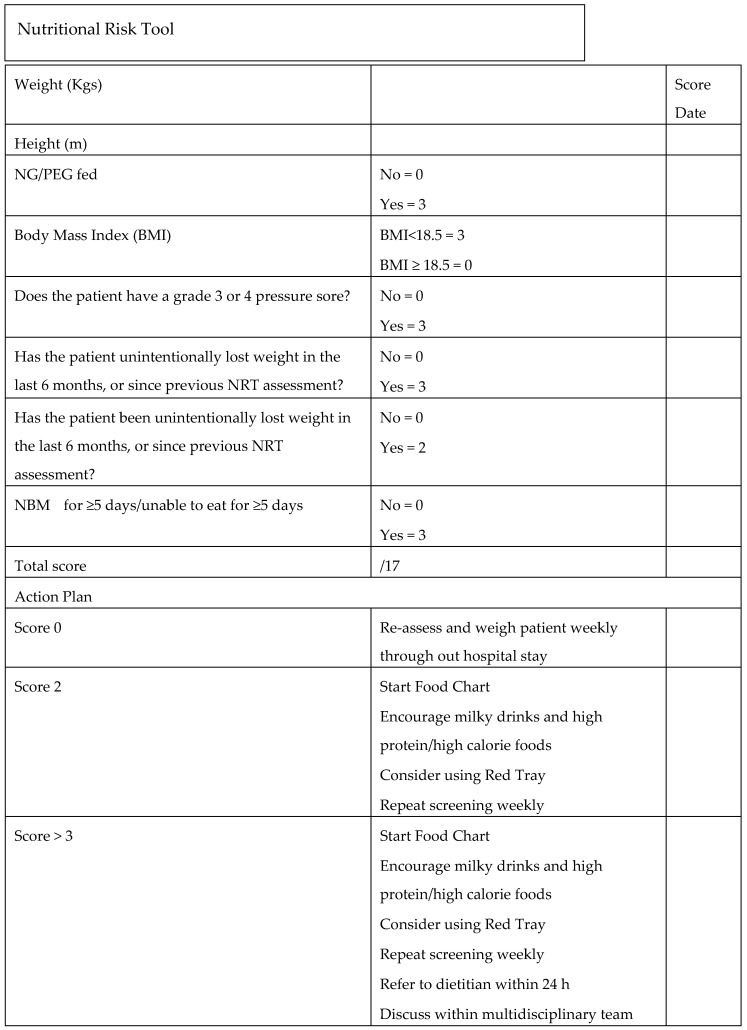
Nutritional Risk Tool (provides Nutritional Risk Score).

Sarcopenia was screened for using the SARC-F tool. A score of ≥5 was taken as a significant level on the SARC-F as per previous work [10]; frailty was assessed using the clinical frailty score (CFS), where a score of 1−4 indicates not frail or mild frailty, 5–6 moderately and 7–8 severely frail or 9 terminal care; nutrition using the Nutrition Risk score (Figure 1); dysphagia using the 4QT, where a score >1 on the 4QT indicates a swallowing problem (Box 1).

A subset of 40 patients had their dominant hand grip strength assessed (L.M.) using a Jarrod dynamometer. Patients were asked to grip the dynamometer in their dominant hand, with the arm at right angles and the forearm resting on a hard surface and squeeze as tightly as possible for 5 s. The peak value was identified and then the assessment was repeated such that an average of three maximal effort squeezes could be calculated.

Box 1 **4QT Swallow screen.**

Do you cough and choke when you eat and drink?

Does it take longer to eat your meals than it used to?

Have you changed the type of food that you eat?

 

Does your voice change after eating/drinking?

 

Total (Score 1 for each positive answer).

A score >0 may indicate the presence of dysphagia/ swallowing problems.

## 3. Statistical Analysis

Statistical calculations were undertaken using the statistical functions of Microsoft^®^ Excel.

To investigate the associations between frailty, sarcopenia, nutrition and dysphagia, linear regression statistics were used to calculate the Pearson’s correlation coefficient (Table 1 and Figure 3). Multiple regression analysis was undertaken to look at the relationship between nutritional risk, sarcopenia, frailty, and age with dysphagia.

## 4. Results

**Figure 2:**
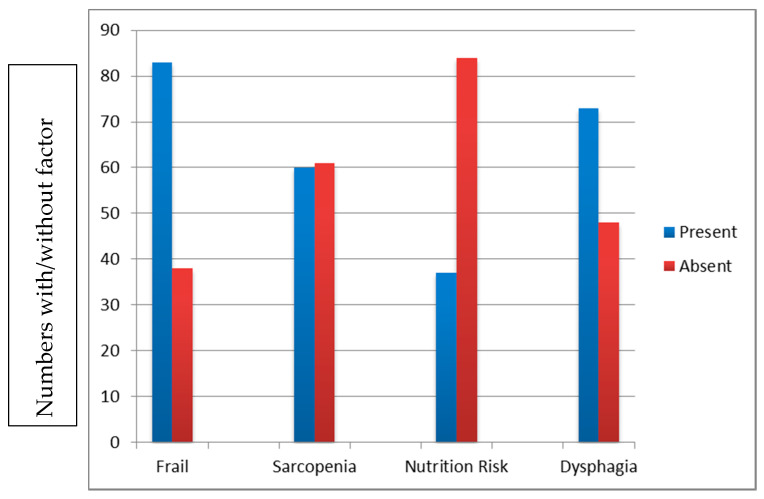
Incidence of each of the quartet in a convenience sample of older adults admitted to acute frailty and medical wards in our study.

In total, 135 people agreed to take part. Thirteen were <65 years of age and have been excluded from the analyses. The data from 122 people were analysed. The mean age of the participants was 82.93 years (65–99 years), and 52.46% (64) were female. Overall, 111 of the 122 (91.0%) people taking part reported the presence of at least one of the quartet. The median CFS was 5 (1–9), with 84 (68.85%) having a score of ≥5 (moderate or severely frail); the median SARC-F was 5 (0–10), with 64 (52.45%) people having a score of ≥5; the median NRT was 0 (0–8), with 33 (27.05%) scoring ≥1, suggesting concerns from a nutritional point of view. Seventy-seven (63%) people reported difficulty with swallowing/dysphagia (4QT ≥1). (Figure 2).

Of the 29 that only had one of the quartet; 31.03% (9/29) were frail; 6.89% (2/29) had sarcopenia; 13.79% (4/29) were nutritionally at risk, and 51.72% (15/29) reported problems with their swallowing. A total of 16 patients (13.11%) had all four of the quartet and could be described as the “most vulnerable” of all who took part.

The subset of 43 patients (53% female, mean age = 83.4 years; range = 74–99 years; median frailty score CFS = 5) completed the HGS at the time of the other assessments. There was a statistically significant relationship between HGS and SARC-F (*r* = −0.4261, *p* < 0.0001) and CFS (*r* = 0.46421, *p* < 0.0001). No relationship was demonstrated between the HGS, dysphagia and nutritional status.

**Figure 3:**
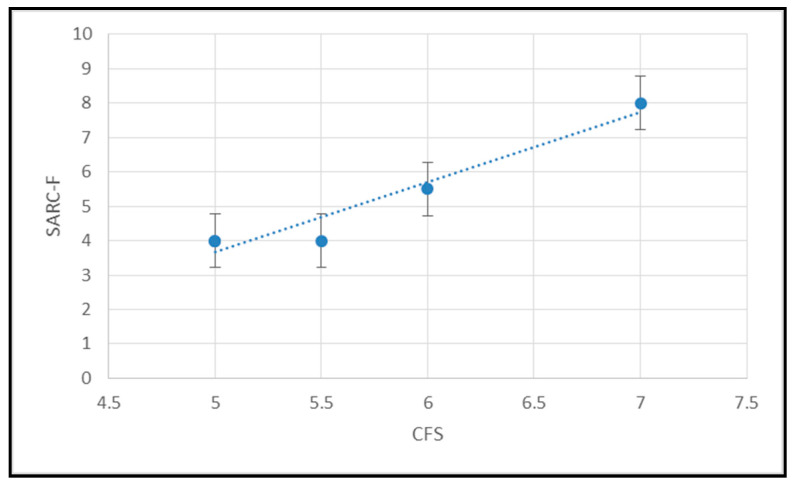
The relationship between SARC-F and CFS (Values are Means ± SEM).

The correlation coefficients are documented in Table 1. No correlation was found between frailty or sarcopenia and nutritional status (NRT); nor dysphagia and sarcopenia. There was a strong correlation between sarcopenia and the clinical frailty score (Figure 3, Table 1) and dysphagia had a strong relationship with nutritional risk and clinical frailty scores (Table 1).

Multiple logistic regression analysis was conducted to investigate the relationship between frailty, sarcopenia and nutritional risk with dysphagia. The results were that nutritional risk and frailty were related to dysphagia but sarcopenia, and age were not (*f*(1119) = 14.872; *p* < 0.00001), *γ* = 0.0595 + 0.2702 × *X*_1_ + 0.1568*X*_2_. Where *X*_1_ is nutritional risk and *X*_2_ is frailty; *γ*, the dependent variable is dysphagia. 

## 5. Discussion

A convenience sample of 122 older people was recruited from the acute frailty and medical wards. The prevalence of one or more of the four main factors that we measured (frailty, sarcopenia, nutritional risk and dysphagia) was 91.0%. This is the first time that the prevalence of, and relationships between, all four factors has been reported. Therefore, it is difficult to compare our findings with previous studies. However, Shiozu et al., reported that those with sarcopenia were more likely to have dysphagia, be undernourished and have decreased functional abilities [19]. Moreover, frailty has been shown to be positively related to age and mortality, after 8 years follow up [20].

In 2014–2015, 20% of hospital admissions involved people over 75 years of age and between 22% and 40% were frail [21]. Wallis et al., found that about 60% had a CFS ≥5 on admission [22]. Frailty is dynamic and depending on clinical approach it may recede or progress [23]. Joosten et al., in a smaller cohort, determined the prevalence of frailty using the Cardiovascular Health Study. Here, 40% were frail and ≈58% were pre-frail [24]. Other studies have suggested that the prevalence of frailty is as high as 80% in hospitalised older adults [25,26]. We have found that nearly 70% of our cohort were assessed as having a preadmission CFS of ≥5, which concurs with the previous studies.

Sarcopenia is frequently overlooked and not assessed in the acute hospital setting. Community-based studies have reported the prevalence of sarcopenia in older men to be about 7% [27]. Morandi et al., in an acute hospital setting, found that about 50% of their participants (mainly female) had a 75% chance of sarcopenia [28]. This study found that about 53% were predicted to be sarcopenic using the SARC-F. Sarcopenia is commonly associated with frailty. In this study there was a correlation between the two. Sarcopenia has often been assessed using handgrip strength [29]. Perez-Zepeda et al., in a small longitudinal study, found that 40% had evidence of sarcopenia using handgrip strength as an indicator [29].

Poor nutrition in hospitalised patients is not a new phenomenon. McWhirter highlighted concerns in 1995 [30]. Somachi et al. [31] documented that 33% of hospital admissions were undernourished and suggested that two thirds will decline if not managed. Axelsson, studying stroke patients, noted that the longer someone was in hospital the more likely they were to become malnourished [32]. In England, the CQC (regulatory body) found that 50% of hospitals were not following national guidelines or supporting people to maintain their nutritional status [33]. In 2013, the Interdisciplinary Alliance published a “call to arms” [34] to address this problem. Agarwel et al., in a large study of 3122 admissions, found that 32% were malnourished and 23% ate less than 25% of the food offered to them [35]. This was more common in patients older than 65 years. In relation to under-nutrition in community dwelling older people, Schilp et al. (2012) reported rates of 35% in a sample of Dutch adults who were receiving home care [36].

Swallowing is complex and requires synchronization of swallowing and breathing. The suprahyoid muscles, laryngeal and pharyngeal muscles receive constant input from the respiratory centres and may synchronize with the diaphragm during swallow. Dysphagia is common in older people with different rates of prevalence reported in different clinical and social settings [15,37,38,39]. Between 2005 and 2006, of people admitted to hospital in the USA, 271,983 (0.35% of 77 million admissions) had dysphagia [40]. The same study reported that, in those aged 75 years and over, 0.73% had dysphagia [40]. In a study of patients with dementia, the prevalence of dysphagia was reported to be 55% of those admitted to an acute hospital [14]. Carrion et al., found that ≈47% of older people who were ≥70 years admitted to an acute geriatric unit in Spain had oropharyngeal dysphagia on clinical assessment, using the V-VST [13]. Despite the fact that the prevalence of dysphagia can be quite high, there is frequently a failure to screen for it [41].

However, many studies have found no association between handgrip and dysphagia, except in the case of heart failure [42]. Maeda and Akagi reported a prevalence of sarcopenia of ≈77%, and prevalence of dysphagia of 30% in a cohort of 224 [43]. A more recent paper found an association between frailty and dysphagia by using the Timed up and Go test. Again, this study found no relationship between HGS and reported dysphagia. On the other hand, Yoshimura found an association between sarcopenia and dysphagia in a convalescent cohort [44]. The lack of clarity regarding the relationship between muscle strength, frailty and dysphagia may be that handgrip strength is not specific to the strength of the muscles involved in swallowing. A more appropriate muscle group for assessment of swallowing-related strength may be the suprahyoid and masseter muscles. Recent work has shown that there is a relationship between jaw strength (mmHg) and HGS (psi) with a reported correlation of *r* = 0.556 (HGS in pounds per square inch = 0.59 × jaw strength in millimetres of mercury + 44). Further work is required to validate this relationship. Studies have shown that sarcopenia is related to functional outcome and food intake [45].

We know that frailty, sarcopenia, nutritional risk and dysphagia are all associated with longer length of hospital stay, increased morbidity and mortality and an increase in healthcare costs. One study suggested that the presence of dysphagia added ≈40% to healthcare costs [46]. Garcia-Nogueras et al. [47] found a strong association between health costs and frailty allowing for comorbidities. Brock et al., using Fried’s five frailty criteria, showed that frailty scores ≥3 (irrespective of age) were associated with increased healthcare costs of USD 3659 compared to USD 642 for non-frail patients and this remained the case for at least one year [48,49]. Westmark et al., found that patients admitted with a diagnosis of dysphagia cost USD 4282 more than patients without dysphagia [50]. The presence of sarcopenia increased hospitalisation costs by USD 1240 [51]; in the US, sarcopenia is responsible for 1.5% of direct total health care costs, or USD 900 per person per year [52].

Poor nutritional state, sarcopenia, frailty and any underlying cause of dysphagia are associated with a chronic inflammatory state and a suppressed immune system [53,54]. All four factors are associated with a decline in function and increased mortality [40,55]. Sixteen patients (≈13%) in this study were frail, sarcopenic, nutritionally at risk, and reported dysphagia. These are potentially a very vulnerable group, further work is required to investigate this. Apart from a nutritional score, many acute units do not routinely screen for frailty, sarcopenia and dysphagia.

This study has shown that a high percentage of people admitted acutely to hospital are at risk of at least one of the four factors we have highlighted in this study (frailty, sarcopenia, dysphagia and nutrition). It would therefore appear reasonable to expect that, where one was found, at least one of the others will often be present. If someone cannot swallow safely, then they are at risk of malnutrition. This, in turn, results in the loss of muscle mass and poor exercise tolerance and subsequent frailty. It should be a requirement for acute medical/frailty units to screen all older people for frailty, sarcopenia and dysphagia [56] as well as nutrition, as intervention may well influence outcome.

Intervention is potentially complex and needs to be multidisciplinary [34]. It is essential if we are to limit the harm caused and the gradual decline in dependency (including long hospital stays and community care) and death in these older patients who are admitted to acute wards. Unfortunately, despite the simplicity of assessment, frailty, sarcopenia, undernutrition, and dysphagia [34,40] are missed, or not even screened for, in the majority of older people who are admitted to acute hospital wards.

## 6. Limitations

This study is limited in that all the instruments used were screening tools. Formal swallowing assessments by speech and language therapists, bio-impedance and detailed nutritional assessments were not undertaken. This may have resulted in an under-estimation of the scale of the problems.

We have not included the relationship which cognitive decline or dementia would have with the “Older Adult Quartet”. Poor cognition can result in a high frailty score with a low sarcopenia score. Significant cognitive impairment would cause difficulty in completion of all the screens. The inclusion of people with significant cognitive concerns should be included in future work.

This is a single site convenience study, limited to a single site utilizing routinely collected clinical data and not a formal research study.

## 7. Conclusions

Frailty, sarcopenia, nutritional risk and dysphagia are common in older people. This supports the presence of an “Older Adult Quartet”. Further work is required to look at the interdependency within the “Older Adult Quartet”, and their relative contributions to outcome.

## Figures and Tables

**Table 1:** Correlation coefficients for indices of frailty, sarcopenia, nutrition and dysphagia (the older adult quartet).

	NRT	SARC-F	CFS
NRT	x	x	x
SARC-F	0.0991 (NS)	x	x
CFS	0.0005 (NS)	0.6111 (*p* ≤ 0.00001)	x
4QT	0.3842 (≤0.0001)	0.1424 (NS)	0.2308 (*p* ≤ 0.0109)
	(1)		
